# COVID-19: Mechanisms of Vaccination and Immunity

**DOI:** 10.3390/vaccines8030404

**Published:** 2020-07-22

**Authors:** Daniel E. Speiser, Martin F. Bachmann

**Affiliations:** 1 Department of Oncology, University Hospital and University of Lausanne, 1066 Lausanne, Switzerland; 2 International Immunology Centre, Anhui Agricultural University, Hefei 230036, China; 3 Department of Rheumatology, Immunology and Allergology, Inselspital, University of Bern, 3010 Bern, Switzerland; 4 Department of BioMedical Research, University of Bern, 3008 Bern, Switzerland

**Keywords:** SARS-CoV-2, COVID-19, nucleic acid tests, serology, vaccination, immunity

## Abstract

Vaccines are needed to protect from SARS-CoV-2, the virus causing COVID-19. Vaccines that induce large quantities of high affinity virus-neutralizing antibodies may optimally prevent infection and avoid unfavorable effects. Vaccination trials require precise clinical management, complemented with detailed evaluation of safety and immune responses. Here, we review the pros and cons of available vaccine platforms and options to accelerate vaccine development towards the safe immunization of the world’s population against SARS-CoV-2. Favorable vaccines, used in well-designed vaccination strategies, may be critical for limiting harm and promoting trust and a long-term return to normal public life and economy.

## 1. Introduction

The COVID-19 pandemic holds great challenges for which the world is only partially prepared [1]. SARS-CoV-2 combines serious pathogenicity with high infectivity. The latter is enhanced by the fact that asymptomatic and pre-symptomatic individuals can transmit the virus, in contrast to SARS-CoV-1 and MERS-CoV, which were transmitted by symptomatic patients and could be contained more efficiently [2,3]. To limit the damage of COVID-19, primary efforts focus on confinement, with physical distancing and multiple further measures preventing infection [4,5]. At present, many investigations aim at defining optimal strategies to limit viral transmission while simultaneously permitting business and social activities [6]. Scientific insights and understanding of the biological mechanisms of the virus and its capability to spread are primordial. Built on this knowledge, the practical strategies may have at least three priorities: firstly, to continue hygiene measures and physical distancing; secondly, to maximize the viral monitoring, both geographically and in time, to focus viral containment locally and limit transmission wherever and whenever possible; thirdly, to rapidly increase the immunity of the world’s population.

The diagnosis of SARS-CoV-2 infection is achieved through detection of viral RNA from a nasal pharyngeal swab or saliva, by nucleic acid tests (NATs) or tests that detect viral protein antigens [5,7]. In infected individuals, the results are only positive for a relatively short time window, on average until the 14th day after symptom onset [8]. Furthermore, a positive NAT result does not allow scientists to conclude whether the affected person is or will become immune. Therefore, serological tests are needed, as they can detect the various types of antibodies in the blood that persist for months or even years.

**Figure 1:**
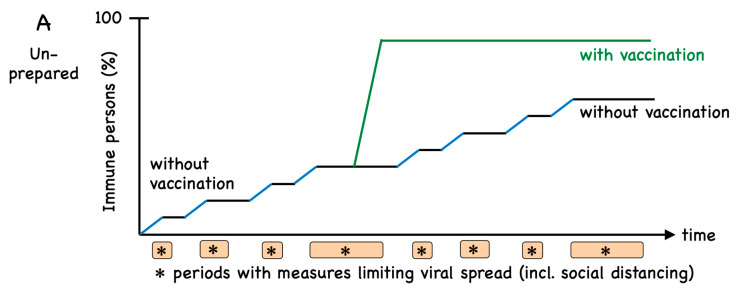

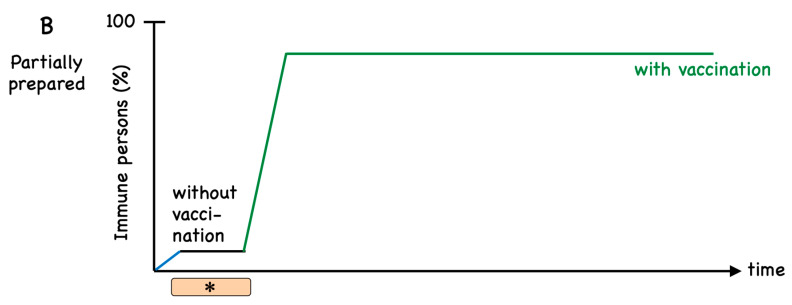
Model of immunity induced by infection and vaccination. (**A**) The world was poorly prepared for the first wave of SARS-CoV-2 spread (blue parts of the curve) [9]. Installed measures for limiting viral spread (*) largely halted further infections (black). Subsequent relaxing of these measures may lead to new waves of viral spreading. Even if this happens several times, it may take significantly more than one year until the majority of individuals become infected and immune. Once available, vaccination (green) will more rapidly induce immunity in a critical percentage of the population, which is necessary for herd immunity. The model scenarios shown are based on current evidence and the assumption that infection and proper vaccines induce immune responses that often protect from (re-) infection. However, there is increasing evidence that immunity to SARS-CoV-2 may only be temporary. This notion is not considered for this Figure because the quantitative importance of waning immunity remains still unknown. (**B**) In case of the emergence of a novel pathogen, vaccination may start much earlier provided one is prepared, i.e., has ample experience with this family of pathogens, enabling rapid vaccine development and production. Unfortunately, this was not the case for SARS-CoV-2. Not shown is the most favored scenario in which the population is previously vaccinated against a given pathogen, precluding viral spread, which is fortunately the case for immunity to many childhood diseases. Additionally, not shown is the in-between scenario in which a vaccine is available but larger parts of the population are not (yet) vaccinated, which can then be readily achieved.

The world can probably not afford to let the majority of citizens undergo SARS-CoV-2 infection, as the overall burden would be enormous. Current data indicate that a pandemic COVID-19 outbreak infects only low percentages (usually in the single digit range) of the population, at least in countries that take effective measures against viral spread [9]. To avoid pandemic propagation, the reproduction number Ro (viral transmission) must remain below 1, meaning that each infected person transmits on average to <1 additional individual, a key aim for continuously reducing case numbers. In contrast, a reproduction number Ro of >1 means a highly unfavorable exponential increase of new infections. Spontaneous disappearance of the virus is unlikely. Additional outbreaks are expected when the safety measures are abandoned (Figure 1A). It may take significantly longer than one year until the majority of the population acquire immunity through infection. As discussed further below, it will be important to determine to which degree natural infection induces immunity and for how long it can protect from re-infection.

The immune response to the SARS-CoV-2 involves innate immune activation and antigen-specific responses of B and T cells [10]. Protection from viral infection is mainly achieved by virus-neutralizing antibodies, a principle that applies to the vast majority of viral infections to which humans acquire robust immune protection due to infection or vaccination. It is urgent to develop vaccines aiming at the induction of protective immune responses, primarily through virus-neutralizing antibodies specific for SARS-CoV-2. Although at least 1–2 years are required to make effective vaccines available globally, vaccination may still be the most rapid and economical strategy to achieve widespread immune protection (Figure 1A). So-called “herd immunity” is reached when a critical percentage of the population has become immune, leaving the virus only limited local chances to circulate. This may be the case when >90% of persons are immune. However, broad immunization is already very helpful as soon as “only” approximately 60–70% have become immune, because relatively simple measures against viral spread will then be sufficient to contain the virus. In case of future outbreaks of newly emerging microbes belonging to well-studied pathogen families, previously established experience will accelerate the development and use of vaccines, allowing us to reach herd immunity more rapidly (Figure 1B). Several organizations, including WHO, the Coalition for Epidemic Preparedness Innovations (CEPI) and the global Vaccine Alliance GAVI, are increasing preparation efforts for future pandemics. The lessons learned from SARS-CoV-2 will certainly support this development which, however, still remains very challenging [1].

Here, we review the mechanistic understanding of immunity and vaccination against SARS-CoV-2. We discuss vaccine target antigens and current vaccine candidates in preclinical and clinical testing. We also focus on the development and validation of precision serology to support preclinical and clinical evaluation of vaccine candidates and research on immune responses induced by natural infection. The basic and applied knowledge acquired during the last few decades is highly useful to design promising vaccines with increased likelihood of inducing protective immune responses and avoiding adverse effects and harmful disease enhancement. Now, probably more than ever, the world depends on rational effective strategies built on robust scientific evidence.

## 2. Vaccine Development

Vaccine development has started at a strongly accelerated pace already, shortly after the beginning of the SARS-CoV-2 outbreak [11,12,13]. At the time of finalizing this review, there are already more than twenty vaccines being tested in clinical trials. The WHO is publishing a regularly updated list of the vaccines in development [14]. As a specialist in the advance of epidemic vaccines, CEPI has organized a global consultation committee which helped to launch the COVID-19 Vaccine Development Taskforce, focusing on vaccine manufacturing and financing, in collaboration with GAVI and the World Bank [15]. Very useful comments on COVID-19 vaccines are regularly published in the scientific literature [16,17,18,19]. The knowledge gained through previous coronavirus outbreaks provides a favorable scientific basis for vaccine design (Box 1)—for example, by helping to identify potentially protective epitopes, the Achilles heels of a virus. However, the fact that SARS-CoV-1 was spontaneously eliminated from the human population, and MERS-CoV was largely controlled without the need for large-scale pharmaceutical interventions, led to a drastic reduction in research funding during the last decade, severely limiting further research and vaccine development. Therefore, there is currently only limited experience on coronavirus vaccination; the first human coronavirus vaccine has yet to be approved.

In view of the urgency of making vaccines available for billions of people, one must primarily focus on vaccines that can be produced in massive amounts and for which the production knowhow and facilities are available or can be built rapidly [20].

Vaccines can be based on whole viruses (live-attenuated or inactivated), viral vectors, nanoparticles or virus-like particles, subunit components, proteins/peptides, RNA, DNA or live cells. The first vaccine trial against COVID-19 was started in China on February 15, 2020 (Table 1), using dendritic cells that are genetically modified with structural and enzymatic proteins of SARS-CoV-2. A second trial, also in China, was done with a similar vaccine, complemented by the infusion of antigen-specific T cells. While both of these vaccines are tested therapeutically in COVID-19 patients, most other vaccines are tested in healthy volunteers. In the US, the first trial was launched in March 2020, using lipid nanoparticle encapsulated mRNA encoding the spike (S) protein, sponsored by Moderna and the National Institute of Health [21]. In early April 2020, a DNA vaccine trial was initiated with a plasmid encoding the S protein, sponsored by Inovio Pharmaceuticals and CEPI. Since mid-April 2020, several vaccines consisting of inactivated SARS-CoV-2 virus have been tested in China [22]. The first viral vector COVID-19 vaccine was developed at the University of Oxford, UK. It is based on a chimpanzee adenovirus and encodes the S protein [23], and it is now in phase 2/3 testing. A similar vaccine is based on adenovirus-5. After promising results in phase 1 in Wuhan, China [24], this vaccine has also moved forward, to a phase 2 trial (Table 1).

Box 1 **Vaccines against SARS-CoV-1 and MERS-CoV.**

Currently there is no vaccine available against these two viral threats [25,26]. Nevertheless, several vaccine candidates against SARS-CoV-1 have been developed based on VLPs, DNA, proteins and viruses (inactivated, live-attenuated, recombinant vectors) [27,28]. While the majority were characterized preclinically, only a few were tested in phase 1 clinical studies [29,30]. Against MERS-CoV, vaccines have been developed based on inactivated virus, DNA and protein, generating preclinical data [31] and phase 1 trial results [32]. For both diseases, larger studies to determine whether the vaccines could protect from natural infection have not been performed.

A study of non-human primates vaccinated with an inactivated virus showed high levels of neutralizing antibodies, high levels of protection and no unfavorable disease enhancement [33]. In the past, some inactivated coronavirus vaccines have been shown to increase disease severity in animals [34], as outlined below in the section on disease enhancement. Attention must be paid to the high mutation rate (e.g., in the S1/S2 junction) which occurs in vitro during the production process of inactivated viruses, requiring careful selection of appropriate vaccine strains. Attenuated viruses may be promising, based on the long history of delivering successful vaccines [35,36] and novel genetic techniques increasing the likelihood to create better vaccine strains [37]. Nevertheless, it takes considerable time to identify strains with the right balance between sufficient attenuation and induction of adequate immune responses. Furthermore, it will be highly demanding to produce sufficient amounts of biosafety level-3 viruses to meet the high global needs. An attractive possibility may be to use inactivated attenuated viruses, as the latter may be more easily grown than the wild-type virus.

Nanoparticles and virus-like particles (VLPs) have delivered successful vaccines. They can be engineered to display epitopes of foreign viruses on their surface, rendering those epitopes highly immunogenic. Molecules that stimulate innate immunity can be encapsulated within VLPs to enhance immune responses and trigger favorable T helper type 1 (Th1) polarized immune responses (type 1 immunity) rather than potentially disease enhancing Th2 polarization [38].

The clinical trials that are now urgently needed to evaluate the COVID-19 vaccine candidates serve to determine optimal vaccine dose and scheduling and whether multiple booster vaccinations are required. Usually, more robust and long-term immunity can be induced by sequential vaccinations, possibly necessary for those with expected weak immune responses, such as in elderly or immune deficient individuals [39]. The clinical studies should also reveal whether the vaccine candidates induce unwanted adverse effects such as local skin toxicity or fever and flu-like symptoms. Autoimmune reactions may also occur, possibly with hematological or neurological manifestations [40]. Fortunately, for most vaccines, severe unwanted adverse effects are very rare. Nevertheless, all safety issues must be carefully studied before a vaccine is used widely.

Table 1. Anti-SARS-CoV-2 vaccines in clinical evaluation, registered at clinicaltrials.gov, clinicaltrialsregister.eu and/or chictr.org.cn. At present, 24 and 142 vaccine candidates are in clinical and preclinical evaluation, respectively [14].

The time required for vaccines reaching the wider public also highly depends on regulatory authorities and their flexibility to accelerate the process and vaccine approval, as compared to standard procedures established for less urgent health threats. The WHO roadmap [41] provides corresponding guidelines and support for regulation and ethics and the use of platforms for developing vaccines and therapeutics in the most efficient ways. Furthermore, properly designed clinical trials may greatly reduce the time for clinical development—for example, by testing several vaccines simultaneously in adaptive trials with a low number of shared control groups [11], requiring unusual cooperativity. Ultimately, however, the most efficient and least toxic vaccines will succeed, even if their development and production take longer. For more details on the production, distribution and safety/efficacy assessment of the different vaccine platforms, we refer to the links in Table 1 and to the existing literature [20,42,43,44,45].

## 3. Vaccine Antigens

### 3.1. B Cell/Antibody Targets

**Figure 2:**
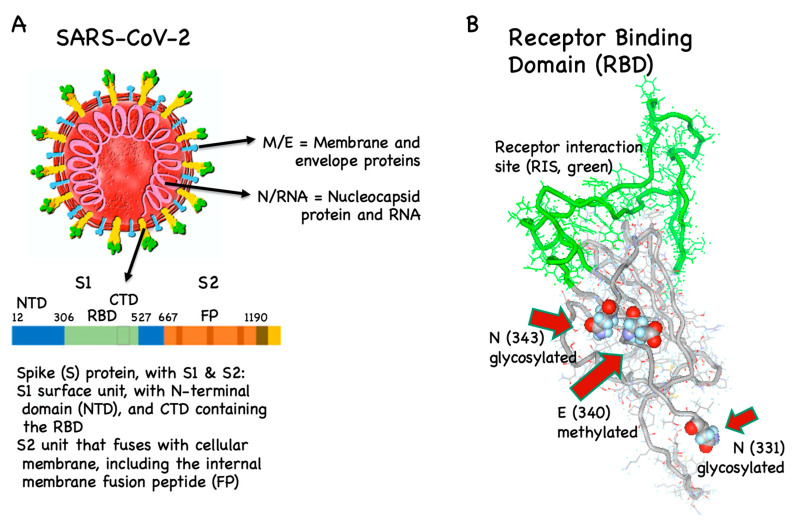
SARS-CoV-2, the spike (S) protein and its receptor binding domain (RBD). (**A**) Coronaviruses have their name because they are decorated by prominent S proteins (yellow/green). It is the only viral protein that interacts with host cells and is the most diverging protein between different coronaviruses, particularly in its receptor binding domain (RBD, green). RBD binds to angiotensin converting enzyme 2 (ACE2, not shown) on the host’s cell surface. The fusion peptide (FP) fuses with the host cell membrane. Specific antibodies against RBD and FP can neutralize SARS-CoV-2 NTD/CTD, N-/C-terminal domains. (**B**) RBD is glycosylated and methylated, which may hinder the induction of neutralizing antibodies. In contrast, the receptor interaction site (RIS, green) is not glycosylated.

Protection induced by currently available vaccines against viruses is primarily based on virus-neutralizing antibodies. Such antibodies usually block the interaction of the virus with its cellular receptor or prevent conformational changes required for fusion of the virus with the cell membrane. The SARS-CoV-1 virus has been studied in substantial detail (Box 2). Recent investigations have shown that the new SARS-CoV-2 virus uses a similar strategy for cell entry [46]. Attachment to host cells takes place via binding of the viral S protein (Figure 2A) to the angiotensin-converting enzyme 2 (ACE2), the viral receptor on host cells. Subsequently, the S protein is primed by host cell proteases, by furin and the serine proteases TMPRSS2 and TMPRSS4, enabling the fusion of viral and cellular membranes and the consequent entry of viral RNA into the host cell [47].

Box 2 **The complexity of the SARS-CoV-1 family and antibody specificities.**

The family of SARS-CoV-1 viruses consists of several groups of strains (comparable to many other viruses) hosted by animals, humans or both [25]. The antibodies are specific for the spike (S), membrane (M), envelope (E), nucleocapsid (N) and further viral proteins. Many of them are strain- or group-specific and thus recognize only some but not all SARS-CoV-1 viruses. Antibodies may be neutralizing, although the majority are not. The neutralizing antibodies are mainly specific for S protein [48,49,50] and, to a minor extent, also 3a protein [51]. Neutralizing antibody epitopes have been found to be highly conserved in several viral strains, indicating that vaccines that elicit such antibodies are protective against multiple strains. These epitopes map primarily to the receptor binding domain (RBD) of the S protein.

S protein interaction with ACE2 is well described for both SARS-CoV-1 and -2 and relies on a particular domain within the S protein, the so-called receptor binding domain (RBD). Indeed, most antibodies capable of neutralizing coronaviruses are directed against RBD [50,52] (Box 2). Hence, the primary immune mechanism of avoiding infection is through blocking viral attachment to ACE2. Therefore, generating a vaccine inducing antibodies against RBD is the strategy used by the majority of COVID-19 vaccine candidates [53]. It has recently been shown that RBD is glycosylated and methylated. Generally, such posttranslational modifications are difficult to reproduce in vaccines, meaning that vaccines may display (slightly) different epitopes than the virus. Consequently, the antibodies induced by the vaccines may potentially be cross-reactive and non-protective. Interestingly, however, the receptor interaction site (RIS) directly binding to ACE2 is not glycosylated, indicating that this RIS may potentially be an ideal vaccine candidate [54,55] (Figure 2B).

The second most frequent choice is to use the whole S1 subunit. Other vaccine manufacturers use the full-length S protein [32] and/or the fusion peptide (FP), which is part of the S2 unit and fuses with the cell membrane and therefore also has neutralizing epitopes [56,57]. The latter is not the case for the N-terminal domain (NTD) of the S protein and the membrane (M), envelope (E) and nucleocapsid (N) proteins, all of which are not directly targeted by the current vaccine candidates [58], also because of the risk of inducing disease enhancing antibodies.

Vaccine antigens may be used in the form of protein or peptides. A recent study has shown that a SARS-CoV-2 S1-Fc fusion protein readily induced neutralizing antibodies in non-human primates [59]. Proteins and peptides may be rendered more immunogenic by formulating them with strong adjuvants. Another strategy is to display vaccine antigens on VLPs, which are often highly immunogenic. Zha et al. have shown that RBD-VLPs efficiently induced SARS-CoV-2-neutralizing antibodies in mice [60]. Further options are to insert RBD into viral vectors or DNA or RNA. A potential challenge is that the induction of neutralizing antibodies depends on antigen display in the correct conformation, which is not guaranteed when a protein or peptide is expressed and displayed in isolation at the site of injection. This may be easier to achieve with the full S protein. However, S protein vaccination may induce non-wanted antibodies in addition to the neutralizing ones directed against RBD [61]. Therefore, provided that one succeeds in constructing a vaccine displaying RBD or even only the RIS in the proper conformation, RBD or RIS may be preferable to the whole S protein.

The most obvious isotype to be induced by a COVID-19 vaccine is IgG, preferably the more protective IgG1 and IgG3 subclasses. However, IgA may also be of importance to reduce infection of mucosa and epithelial cells in the respiratory tract, as well as endothelial cells, which may be widely targeted by the virus. While mucosal immunization at a large scale in a rapid fashion might be difficult, the use of an adjuvant that triggers the production of IgA might be an important consideration. TLR7/8 and TLR9 ligands are good candidates as they potently promote IgA responses [62,63].

### 3.2. T Cell Targets

CD4 and CD8 T cells recognize and react to SARS-CoV-2 antigens [64], contributing to immune protection, particularly by reducing disease severity [65,66]. To some extent, this may also be the case for cross-reactive T cells induced by seasonal coronaviruses [67]. For disease prevention, T cells alone are probably less potent than neutralizing antibodies [68]. Preventive anti-viral vaccines are successful because they induce antibodies that neutralize viral particles in the extracellular space, immediately after body entry and before viruses infect the host’s cells. Importantly, B cell responses and antibody production are strongly promoted by CD4 T helper cells. Therefore, vaccines should simultaneously induce both B cells and T cells.

T cell antigens must be presented by HLA molecules on the surface of antigen-presenting cells and infected cells. As HLA molecules differ in most people due to the huge genetic HLA polymorphism, viral recognition by T cells is based on a very large diversity of antigenic peptide/HLA complexes. Each person has her/his own T cell specificities for those antigenic peptides that bind to her/his HLA molecules. Vaccines containing short antigenic peptides or mini-genes will not work for most people, i.e., for those whose HLA molecules cannot present the respective antigenic peptides. In contrast, vaccination with long peptides or full-length viral proteins or corresponding DNA/RNA are potentially useful for many or all individuals.

CD8 cytotoxic T cells primarily recognize viral peptides that are synthesized within the infected cell. In contrast, protein antigens that are picked up from the extracellular space are poorly presented to CD8 T cells. The one exception to this rule is in small fractions of dendritic cells that are capable of so-called cross-presentation, i.e., the cellular uptake of extracellular protein (especially particulate antigens) and the presentation of processed peptides on HLA class I molecules to CD8 T cells [69]. Cross-presentation is rather slow and often rate limiting, which is a major reason why full-length protein vaccines are inefficient for inducing CD8 T cell responses.

As compared to antibody induction, it is generally more challenging to induce long-term T cell responses through vaccination. Most vaccines are either attenuated pathogens or more often dead/synthetic vaccines which do not replicate in vivo. Yet, substantial microbial replication in vivo is usually required for the induction of strong T cell responses [70]. It is therefore particularly difficult to induce durable CD8 T cells by currently available vaccine technologies. For preventive vaccination, this might not be a major problem, since CD8 T cells are not specialized to prevent infections. Rather, CD8 T cells are important once host cells are infected. Therefore, these cells have their primary role in individuals with established infection.

The induction of CD4 T cell help is often not rate limiting in vaccination, probably because low numbers of these cells are already sufficient for supporting antibody production. Nevertheless, vaccination may fail due to CD4 T cell non-responsiveness. Since T cell help can be provided by CD4 T cells with other specificities by intermolecular help, a smart approach is to supplement vaccines by inserting microbial antigens to which most humans are already immunized [71]. The consequent immune response will be stronger because boosting previously primed and established CD4 T cells is more efficient than priming.

## 4. Disease Enhancement

### 4.1. T Cell-Dependent Disease Enhancement

It is not recommended to vaccinate for T cell responses without also efficiently inducing neutralizing antibodies, because the latter are likely the crucial key effectors, and also because T cells, particularly CD8 T cells, can cause extended tissue damage through their cytotoxicity against infected cells, which is likely increased in the absence of antibodies that neutralize the viruses in the extracellular space. Indeed, pure CD8 T cell responses induced by vaccination may enhance potentially lethal immunopathology [72].

A concrete safety concern is the potential activation of Th2 cells. This was first observed in a clinically tested respiratory syncytial virus (RSV) vaccine, which consisted of inactivated virus and worsened clinical symptoms, with the deaths of two children upon infection with RSV. Disease enhancement was caused by Th2 cell-mediated eosinophilia, a problem associated with vaccines for respiratory vaccines. It is therefore essential to skew the response by vaccination towards Th1 polarization. Indeed, combining inactivated RSV with TLR-agonists reduces lung pathology upon viral challenge in murine models [73]. Experience from SARS and MERS vaccine candidates indicates that this risk exists also for coronavirus vaccines. Immunization with inactivated SARS-CoV-1 [34] caused eosinophilic infiltration in murine models upon viral challenge. While immunization with the viral nucleoprotein may be co-responsible [74], immunization with whole S protein and S protein-based VLPs also triggered the induction of Th2 cells and eosinophilic inflammation upon viral challenge [22]. In contrast, RBD based vaccines induced neutralizing antibodies in the absence of disease enhancement [75]. It should be noted that it may also be advisable to avoid Th17 cells secreting IL-17 upon stimulation, as this may lead to the pulmonary recruitment of neutrophils, contributing to lung damage [76].

### 4.2. Antibody-Dependent Disease Enhancement

**Figure 3:**
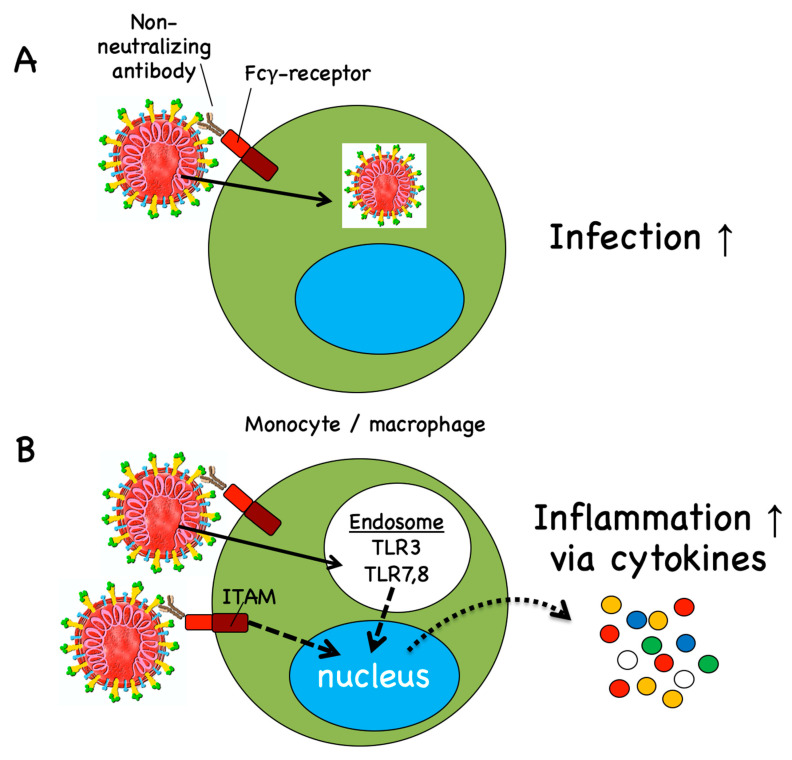
Antibody-dependent enhancement (ADE) of infection and ADE of inflammation. (**A**) ADE of infection occurs through antibodies that mediate Fcγ receptor-mediated viral uptake, leading to increased cellular infection. This mechanism may not apply to human SARS coronaviruses since Fcγ receptor-expressing cells unlikely propagate those viruses in patients. (**B**) ADE of inflammation may occur via Fcγ receptor-mediated virus transfer into endosomes, where viral RNA binds to RNA receptors, triggering inflammatory responses. Alternatively, activatory Fcγ receptors may signal via their ITAM leading to the production of pro-inflammatory cytokines. ADE of inflammation may occur in patients harboring very high viral load in their lungs. ITAM, immunoreceptor tyrosine-based activation motif.

Antibodies can either protect from infection and/or disease severity or be inefficient in doing so. In addition, some antibodies can be harmful. Antibody-mediated enhanced disease may be caused by two different mechanisms. The first is called antibody-dependent enhancement (ADE) of infection, which occurs when antibodies promote viral uptake via Fcγ receptors (Figure 3A), thereby increasing viral infection and pathogenicity [77,78]. ADE of infection is well known for flaviviruses, particularly Dengue virus. The pathogenicity of these viruses occurs via their tropism to macrophages, which can be enhanced by IgG antibodies enhancing viral uptake via Fcγ receptors, increasing cellular infection. Particularly important are antibodies that are cross-reactive to different flavivirus family members. Previous exposure to vaccines or infection-displaying antigens from a different Dengue virus serotype may result in more severe Dengue fever, while infection with the same virus results in protection [79,80]. ADE of infection is preferentially mediated by low-affinity, cross-reactive antibodies and requires the expression of Fcγ receptors by target cells, i.e., the cells that allow viral replication and thus give rise to higher viral burden.

There is no evidence in humans that ADE of infection occurs with SARS-CoV-1 [78]. However, this has been demonstrated in feline infectious peritonitis [81]. Furthermore, the enhancement of hepatitis was observed in ferrets challenged after vaccination with recombinant modified vaccinia Ankara virus expressing the SARS-CoV-1 S protein [82]. Interestingly, similar to Dengue viruses, feline coronavirus infects macrophages, making it more likely that disease severity may increase through ADE-mediated viral uptake via Fcγ receptors. In contrast, human SARS coronaviruses appear to have different tissue tropisms. SARS-CoV-1 infects pneumocytes in the lungs and surface enterocytes in the small bowel [83], both of which do not express Fc receptors. Occasionally, SARS-CoV-1 was also found in lung macrophages; localization was, however, restricted to phagosomes, suggesting that the virus is degraded rather than infecting macrophages [84,85]. Since the two SARS viruses seem to have similar cellular uptake mechanisms [46], COVID-19 is probably not worsened through ADE of infection.

The second mechanism is ADE of inflammation (Figure 3B). Activatory Fcγ receptors have special signaling motifs (immunoreceptor tyrosine-based activation motifs; ITAMs) that may directly mediate immune cell activation. Alternatively, Fcγ receptor-mediated viral uptake into immune cells may promote the production of inflammatory molecules by triggering RNA sensors [86]. These pathways lead to up-regulation of inflammatory cytokines and chemokines such as TNF, IL-6, CCL2 and CCL3 and reduced production of anti-inflammatory factors like IL-10 and TGFβ [74,86]. However, this mechanism is unlikely to be induced by efficient vaccines, because it requires a high viral load, as found in severely ill patients, a situation prevented by vaccine-induced neutralizing IgG antibodies.

Together, these data suggest that efficient and safe strategies of vaccination may be achieved by the preferential usage of antigens that display neutralizing epitopes and the relative avoidance of other epitopes to limit the risk through disease enhancing antibodies. Hence, RBD or RIS alone, perhaps combined with the fusion peptide, could be optimal, as other parts of the S protein and the other SARS-CoV-2 surface proteins could be potentially involved in ADE. Furthermore, vaccines that promote Th1 cell responses are preferable, which can be achieved by using viruses/viral vectors or innate immune stimulators with type 1 polarization capabilities [87,88,89].

## 5. Assays for Measuring Coronavirus-Specific Immune Responses

### 5.1. Serology

Most respiratory viruses induce IgM, followed by IgG and IgA antibody responses. Seroconversion to SARS-CoV-2 infection occurred after 7 days in about half of the patients and by day 14 in nearly all patients [90]. On average, the IgM response may peak 7 to 10 days after infection and the IgG response at about 3 weeks [91]. Already, many serology assays are available for detecting SARS-CoV-2-specific antibodies, some of which have reached sufficient reliability to be suitable for mass testing [7,92]. It is important to validate these assays such that results can be pooled and compared; centralized laboratories may greatly contribute to this. Serological methods may be used to detect (prior) infection or to evaluate possible protection from infection. Tests that indicate prior infection have to be highly specific for SARA-CoV-2 and may include several viral proteins such as nucleoprotein and spike protein to increase sensitivity. A test that discovers IgM antibodies may indicate ongoing infection, while IgG in the absence of IgM may indicate clearance of the virus. IgA antibodies are potentially useful if saliva is to be tested. The major issue of an IgA test is false negativity as IgA levels are often low. Therefore, results may need to be verified by additional serological testing and/or NATs.

**Figure 4:**
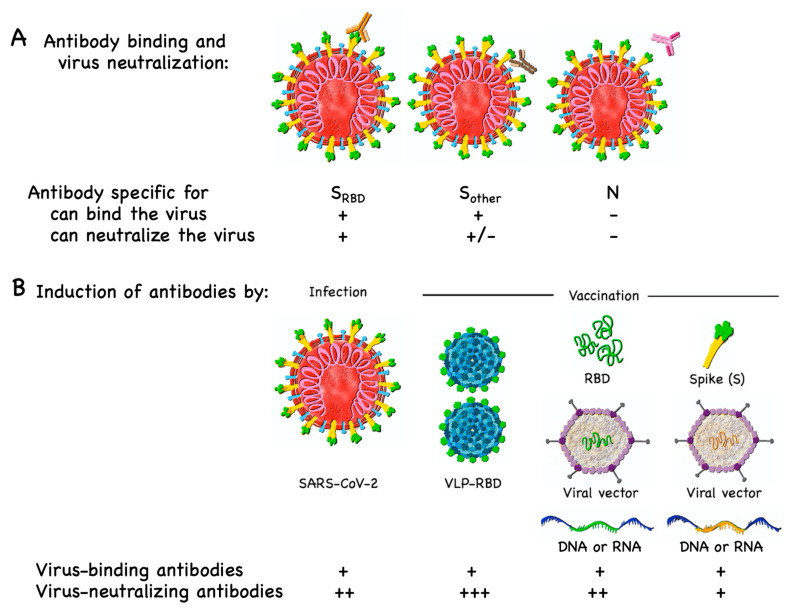
Different types of antibodies and induction of antibodies by infection and vaccination. (**A**) Antibodies (orange or brown) specific for viral surface proteins can bind to SARS-CoV-2, in contrast to antibodies (pink) specific for the viral nucleoprotein (N), which is not accessible in viable viruses. Antibodies (orange) that bind to RBD are likely neutralizing, as they block the attachment of the virus to its receptor (ACE2) on the surface of host cells (not shown). Most antibodies (brown) binding to other moieties of the spike (S) protein (and antibodies binding to envelope or membrane proteins of SARS-CoV-2; not shown) may not neutralize the virus. +, yes; +/- eventually; - no. (**B**) Virus-binding antibodies may be induced by infection or vaccine candidates. Virus-like particles displaying RBD (VLP-RBD) have a high likelihood of inducing neutralizing antibodies, provided that they display RBD (green) in a repetitive and thus highly immunogenic manner. Alternatively, RBD-based vaccines may be produced with RBD peptide, or viral vectors, DNA or RNA encoding RBD. The same vaccine types may incorporate alternative antigens such as the full S protein (yellow), which may differ in the degree of immunogenicity but may also be more likely to trigger virus-binding non-neutralizing antibodies, possibly increasing the risk for antibody-dependent enhancement (ADE). Inactivated and live-attenuated viruses (not shown) are expected to have relatively similar antigenic profiles to wild-type virus. +++, strong; ++ intermediate; + weak.

Unfortunately, most serology tests cannot directly determine whether the detected antibodies neutralize the virus, a mandatory parameter if protection is to be predicted. Upscaling of neutralizing antibody testing is limited because assays that directly demonstrate neutralization of SARS-CoV-2 must be done with the virus itself, which is only safe in highly specialized biosafety level-3 laboratories. Many groups have developed neutralization assays with retroviruses or vesicular stomatitis virus (VSV) pseudotyped with the SARS-CoV-2 S protein, allowing neutralization assays to be done in biosafety level-2 facilities [78,93,94,95,96]. A surrogate for neutralization is antibodies specific for RBD, as most SARS virus-neutralizing antibodies are binding to RBD [52,96,97] (Figure 4A), although a small number of antibodies may neutralize SARS without binding RBD [98] (Box 2). In contrast to tests indicating infection, where false negative responses should be minimized, in assays predicting protection, false positive results should be minimized. As RBD substantially differs between different coronaviruses (binding to different receptors), this domain may also be ideal in terms of virus specificity.

SARS-CoV-2 is known to mutate, bearing the risk that existing neutralizing antibodies may lose their protective power [78]. Even though this possibility cannot be excluded, coronaviruses appear to change relatively slowly [64], in contrast to others such as influenza viruses or HIV-1. Coronaviruses have an RNA proofreading capability [99], supporting the view that members of this virus family depend on a relatively high degree of genetic stability. Indeed, common S protein mutations of SARS-CoV-2 are unlikely to affect antibody epitopes [50]. Thus, antibodies may keep their protective power for a time period long enough to justify vaccine approaches. The major threat of coronavirus evolution may not be the slow adaptation to existing immunity, as known from influenza as genetic drift (requiring yearly new vaccines). Rather, the high danger comes from genetic recombination events in animals infected with multiple viruses, as known from influenza as genetic shift, the typical origin of influenza pandemics [100]. In any case, vaccine development must be accelerated to cope with future challenges, since new coronaviruses or other microbes may threaten the world’s population.

### 5.2. Cytokine Measurements and T Cell Analysis

Assessment of Th2 cytokines such as IL-4/IL-5, or IL-17 as a marker for Th17 cells, may help to evaluate the risk of disease enhancement. Furthermore, measurements of cytokines and inflammatory parameters are important for monitoring patients with (risk of) severe SARS, in which strong cytokine production and inflammatory reactions may be associated with lung damage [101,102]. Increased levels of IL-6, ferritin, D-dimer, LDH and cardiac troponin I were found to be associated with increased disease severity [103]. Since novel therapies must closely consider and target pathogenic mechanisms, novel approaches are aiming at their early detection, hopefully permitting treatment early upon infection so as to prevent progression to severe disease and a lethal outcome.

T cells are infrequently analyzed, and only in specialized laboratories, because viable cells require special and labor intensive laboratory handling [104]. The great variability of peptide/HLA antigens further complicates matters. In general, the presence of CD4 T helper cell responses can be deducted by the demonstration of T cell help-dependent antibody responses which switch to IgG isoforms.

## 6. Immune Responses to Natural Infection Versus Vaccination

There is currently still only little understanding of the relationships between SARS-CoV-2 infection, antibody responses and protection. A central issue is to determine whether vaccine candidates induce immunity. There are serious ethical and technical limitations to challenging vaccinated volunteers with live wild-type viruses with the aim of determining vaccine effectiveness. Alternatively, challenging with seasonal coronaviruses or attenuated viruses could be considered for this purpose, substantially reducing the risk for trial participants. In any case, trials should include volunteers with a high risk of natural infection, such as populations in highly affected regions and healthcare workers.

Based on the current evidence, SARS-CoV-2 infection induces at least some degree of immunity, perhaps even in the majority of individuals [105]. However, immunity might be less sound as compared to immunity induced by SARS-CoV-1 infection, after which about 90% of patients had antibodies still detectable after two years [106]. At three years post infection, IgG antibodies and neutralizing antibodies were detectable in about 80% of patients [107]. SARS-CoV-2 induces milder disease, on average, and is often only limited to the upper respiratory tract, in contrast to SARS-CoV-1, which nearly always affected the lower respiratory tract as well. Re-infection with SARS-CoV-2 has been reported but these cases could also reflect false negative results from viral RNA testing, potential inadequate timing of sampling or reactivation of the virus [108,109]. For MERS, it is unknown whether it induces protective immunity [110].

Interesting insights are offered from studying seasonal coronavirus common cold infections, which occur typically in winter to spring and are usually of mild nature [111]. Indeed, the experimental induction of relatively mild infection with seasonal coronaviruses were followed by short-lived protection in the range of one year [112] or less [113]. More encouragingly, however, both studies demonstrated that protection against experimental infection correlated with increased levels of IgA and IgG antibodies present at the time of inoculation, both for homologous and heterologous viruses. Furthermore, these seasonal coronavirus infections come in waves that may be self-limiting, as in the years 1977–1979, when 90% of individuals had neutralizing antibodies against the 229E strain, presumably reducing infection rates [112].

A reason for the short duration of protection may be that coronaviruses have an interesting strategy to evade neutralizing antibody induction. As discussed previously for adenoviruses [114], SARS viruses may simply dilute the S protein in a sea of other proteins on the viral surface. In this way, the spacing of S proteins becomes too large for optimal B cell activation, which is at a distance of 5–10 nm [115], resulting in suboptimal generation of anti-S neutralizing antibodies. The fact that this protein is long and embedded in the membrane of a large virus may reduce its highly repetitive and rigid display, further reducing its immunogenicity. For these reasons, immune responses against SARS viruses may be dominated by non-protective antibody responses. If these considerations apply, SARS-CoV-2 may not be able to rapidly adapt to strong neutralizing antibody responses as it has evolutionarily never been confronted with that challenge. Hence, COVID-19 vaccines designed to optimally expose the RBD to the immune system for the efficient induction of neutralizing antibody responses could potentially exert un unprecedented pressure on the virus, resulting in a halt of viral spread (Figure 4B).

## 7. Conclusions and Perspectives

A major hurdle is the very limited pre-existing clinical experience with any coronavirus vaccine, increasing the failure risk of COVID-19 vaccine trials and consequent delay. Fortunately, the ongoing multitude of parallel vaccine development may compensate for the experience deficit. Furthermore, there are many basic, translational and preclinical data in coronavirus research, which together with the massive ongoing scientific effort forms a favorable basis for rapid progress.

We suggest that COVID-19 vaccines are promising when they induce large quantities of high affinity neutralizing antibodies and only relatively low amounts of other antibodies and immune responses bearing the risk of disease enhancement. Targeted by most neutralizing antibodies, RBD may be the virus’ Achilles heel. However, it is still only partially possible to predict vaccine efficacy and safety [116]. It remains justified to pursue the development of multiple different vaccine types, also against other target antigens, possibly increasing the likelihood of success.

The large scale use of vaccines inducing neutralizing antibodies is the best option to maximize the percentage of the population with immunity to SARS-CoV-2. It is realistic to achieve herd immunity through vaccination, whereas broad natural infection appears too risky for humans and the economy, unless viral spread induces immunity in much larger fractions of the world’s population than currently known and expected, possibly in countries with less rigorous measures for avoiding viral spread. Due to the urgency, COVID-19 vaccination is given high priority.

Once proven efficient and safe, vaccines should undergo registration to ensure that the world is prepared for current and possible future SARS-CoV-2 outbreaks. At the same time, measures should be put in place, as is the case for influenza virus vaccines, that allow the rapid adaptation of existing vaccine platforms to newly emerging coronaviruses.

## Figures and Tables

**Table 1:** SARS-CoV-2 vaccine candidates in clinical trials.

Vaccine Candidate	Platform, Route of Administration	Target (SARS-Cov-2)	Developer	Trial Phase, Registry Number, Study Start, Link
Synthetic minigene transfected APCs Covid-19/aAPC	Artificial antigen presenting cells (APCs) modified with lentiviral vector, s.c..	Selected conserved structural and protease protein domains	Shenzhen Geno-immune Medical Institute, China	Phase 1/2, NCT04299724, 15 February 2020 http://szgimi.org/en/news.php
Synthetic minigene transfected APCs + cytotoxic T cells LV-SMENP-DC	Dendritic cells modified with lentiviral vector, s.c., plus i.v. infusion of cytotoxic T cells	Viral structural proteins and a polyprotein protease	Shenzhen Geno-immune Medical Institute, China	Phase 1/2, NCT04276896, 24 March 2020 http://szgimi.org/en/news.php
Recombinant adenovirus, Ad5-nCoV	Viral vector, Adenovirus 5, i.m.	Spike protein	CanSino Biologics, China	Phase 2, NCT04341389, 12 April 2020 http://www.cansinotech.com/homes/article/plist/56.html
Recombinant adenovirus, AZD1222	Viral vector (non-replicating) Chimpanzee Adenovirus, i.m.	Spike protein	University of Oxford, UK, & AstraZeneca	Phase 2b/3, 2020-001228-32, 4 May 2020 https://www.ox.ac.uk/news-and-events/for-journalists
Recombinant adenovirus, Gam-COVID-Vac (Lyo)	Viral vector, Adenoviruses 5 and 26, i.m.	Spike protein	Gamaleya Research Institute, Russia	Phase 1, NCT04436471, 17 June 2020 http://gamaleya.org/
Plasmid, INO-4800	DNA, i.d., followed by electroporation	Spike protein	Inovio Pharmaceuticals USA, & CEPI	Phase 1, NCT04336410, 3 April 2020, and Phase 2, https://www.inovio.com/our-focus-serving-patients/covid-19/
Plasmid + adjuvant, AG0301-COVID19	DNA, i.m.	Spike protein	AnGes and Osaka University, Japan	Phase 1/2, NCT04463472, 29 June 2020 https://www.anges.co.jp/en/
Plasmid, GX-19	DNA, i.m.	Spike protein	Genexin Inc., Korea	Phase 1/2, NCT04445389, 17 June 2020 http://www.genexine.com/m62.php?cate=1
Lipid nanoparticle encapsulated RNA, mRNA 1273	mRNA, i.m.	Spike protein	Moderna and Natl Inst Allergy & Infectious Diseases (NIAID), USA	Phase 2, NCT04405076, 25 May 2020 https://www.niaid.nih.gov/clinical-trials/safety-immunogenicity-study-vaccine-covid-19
Lipid nanoparticle encapsulated RNA, BNT162	mRNA, i.m.	Various viral ags (4 vaccine candidates)	BioNTech, Germany, & Pfizer, USA	Phase 1/2, NCT04368728, 29 April 2020 https://investors.biontech.de/press-releases
Lipid nanoparticle encapsulated RNA. CVnCoV	mRNA, i.m.	Spike protein	CureVac, Germany	Phase 1, NCT04449276, 18 June 2020 https://www.curevac.com/covid-19
COVAC1 (LNP-nCoVsaRNA)	mRNA in lipid nanoparticle, i.m.	Spike protein	Imperial College London, UK	Phase 1, ISRCTN17072692, 1 April 2020 http://www.imperial.ac.uk/news
Protein + adjuvant, NVX-CoV2373	Protein subunit vaccine, i.m.	Spike protein and Matrix-M adjuvant	Novavax, USA	Phase 1/2, NCT04368988, 25 May 2020 http://ir.novavax.com/press-releases
Protein + adjuvant, SCB-2019	Protein trimeric subunit vaccine, i.m.	Spike protein, AS03, CpG, alum adjuvant	Clover Biopharma, Australia, GSK, Dynavax	Phase 1, NCT04405908, 19 June 2020 http://www.cloverbiopharma.com/
SARS-CoV-2 inactivated virus, PiCoVacc	Inactivated virus + alum adjuvant	Entire virus	Sinovac Research and Development Co, China	Phase 1/2, 16 April 2020, and Phase 3 http://www.sinovacbio.com/?optionid=754&auto_id=904
SARS-CoV-2 inactivated virus	Inactivated virus	Entire virus	Chinese Academy of Medical Sciences	Phase 1/2, NCT04412538, 15 May 2020 http://english.cas.cn/newsroom/news/
SARS-CoV-2 inactivated virus	Inactivated virus	Entire virus	Sinopharm	Phase 1/2, ChiCTR2000031809, 11 April 2020 http://www.chinacdc.cn/en/
